# Advances in the pathophysiological study of brain development: application of cerebral organoid combined with Spatial omics technology

**DOI:** 10.1186/s13287-025-04885-3

**Published:** 2026-01-23

**Authors:** Jiayi Wang, Zhaokai Sun, Yiran Zhou, Liang Wang, Jing Liu

**Affiliations:** National Joint Engineering Laboratory, The First Affiliated Hospital of Dalian Medical University, Dalian, Liaoning P.R. China

**Keywords:** Spatial multi-omics, Cerebral organoids, Neurodevelopment, Neurological diseases

## Abstract

Understanding the complexities of the human brain development remains one of the most formidable challenges in neuroscience, constrained by the limitations of traditional models and the inaccessibility of brain tissue. The advent of cerebral organoids has provided a transformative in vitro model that closely mimics the early stages of brain development, including the spatiotemporal organization and cellular heterogeneity. Derived from pluripotent stem cells, these self-assembling three-dimensional structures address critical limitations of earlier systems, including species-specific differences in animal studies and the structural constraints of conventional cell models. Over the past decade, cerebral organoids have enabled significant advances in studying neural development, neurogenesis, modeling neuroconnectivity, and investigating neuroregeneration. Meanwhile, high-throughput spatial multi-omics technologies have emerged for decoding molecular and cellular dynamics with spatial precision. These techniques retain the architectural context of biological samples while integrating diverse layers of omic information, providing unprecedented insights into tissue organization and interactions. By addressing the complexity of brain organization and facilitating actionable insights into neurodevelopmental diseases, this integration facilitates high-throughput drug screening, identifies disease-specific targets, and offers a path to novel therapeutic strategies and regenerative solution for future stem cell therapies for pediatric neurodevelopmental diseases.

## Introduction

The human brain stands as a masterpiece of biological complexity, unparalleled by any other organ or system [1]. Its intricate neural networks not only form the foundation of human cognition and behavior but also inspire artificial intelligence systems that strive—albeit imperfectly—to replicate its precision and adaptability [2]. Comprising approximately 86 billion neurons, each forming about 7000 synaptic connections, the brain orchestrates a vast range of functions, from sensory processing to abstract thought and emotion [3, 4]. However, childhood constitutes a critical window for brain development, wherein perturbations during this period can lead to profound and lifelong sequelae. The remarkable complexity of brain development remains unparalleled by any artificial construct or biological model, underscoring the profound challenge in fully elucidating its underlying mechanisms. This inherent complexity likewise renders neurodevelopmental disorders—including developmental delay, primary epilepsy, and autism spectrum disorder (ASD)—among the most challenging conditions to treat [5–7]. These conditions often stem from aberrations in the brain’s fundamental functional and structural units during development, disruptions that are intrinsically linked to its complex architecture. While these disorders impose a immense medical burden on families and society, prevailing treatments largely fail to halt or reverse the pathological course, providing merely symptomatic management. Confronting this challenge requires a deep, mechanistic understanding of disease pathogenesis. Yet, progress in this area is fundamentally constrained by the lack of robust, physiologically relevant models that accurately recapitulate human brain development. The brain’s unparalleled complexity is characterized by its dynamic cellular interactions, hierarchical spatial organization, and extraordinary functional diversity—attributes that are difficult to replicate in experimental systems [8]. Compounding these challenges is the inaccessibility of live human brain tissue for research, due to ethical constraints. Animal models have historically been indispensable for investigating central nervous system (CNS) biology, disease mechanisms, and neuroanatomy [9, 10]. These models have yielded foundational insights and enabled invasive studies that are impossible in humans. However, inherent differences in brain structure, development, and function between species significantly limit their ability to accurately model human-specific features, particularly those underlying advanced cognitive processes. These limitations highlight the urgent need for innovative tools that can capture the brain’s extraordinary complexity and facilitate the development of research progress in the physiology and pathology of neurodevelopment in children.

The emergence of cerebral organoid technology represents a transformative breakthrough in overcoming longstanding challenges in human brain research, fundamentally shifting studies of human-derived samples from a linear perspective to a multidimensional approach [8, 11, 12]. Derived from human pluripotent stem cells, cerebral organoids are self-organizing, three-dimensional structures that closely mimic key aspects of human brain development, including cellular lineage specification, spatial organization, and developmental trajectories [13, 14]. Unlike conventional models, cerebral organoids are capable of undergoing complex, ordered structural maturation in vitro, mirroring the dynamic processes of brain development in vivo [15–17]. This remarkable capability enables researchers to study the physiological and pathological progress of children’s brain development processes in real time, offering unprecedented insights into processes that were previously inaccessible with traditional approaches [18]. By leveraging patient-derived induced pluripotent stem cells (iPSCs), researchers can create personalized organoids that replicate the genetic and pathological characteristics of individual patients. For instance, studies have demonstrated how organoids derived from patients with schizophrenia capture disease-specific cellular phenotypes and neurodevelopmental mechanisms, providing a powerful platform for understanding these complex conditions [19, 20]. This personalized approach circumvents the ethical and logistical barriers associated with the use of live human brain tissue, while simultaneously offering a model that is far more relevant than animal systems for studying human-specific brain biology and pathologies. This capability makes them invaluable for investigating children neurodevelopment, disease mechanisms, and therapeutic responses.

While cerebral organoids have significantly advanced our ability to model human brain development and disease by replicating its cellular diversity and dynamic processes, their full potential relies on the development of robust analytical tools that can decode the brain’s extraordinary structural complexity and high-dimensional information processing. The brain is an organ where form and function are inseparably linked—its intricate architecture and precise spatial relationships are fundamental to its ability to perform complex tasks. Current single-cell multi-omics technologies have provided valuable insights into cellular heterogeneity and lineage tracing but disrupt native spatial relationships during tissue dissociation [21]. This process not only results in the loss of critical positional information but may also induce ectopic gene expression, complicating the identification of true cell states and interactions. To overcome these challenges, spatial multi-omics has emerged as a transformative technology [22]. By integrating molecular profiling with precise spatial localization, spatial multi-omics preserves the three-dimensional structure of tissues, maintaining native intercellular interactions and positional relationships. When applied to cerebral organoids, this approach enables researchers to map molecular dynamics, uncover novel spatial patterns, and analyze cell-cell interactions within an intact architectural context [23, 24].

**Fig. 1:**
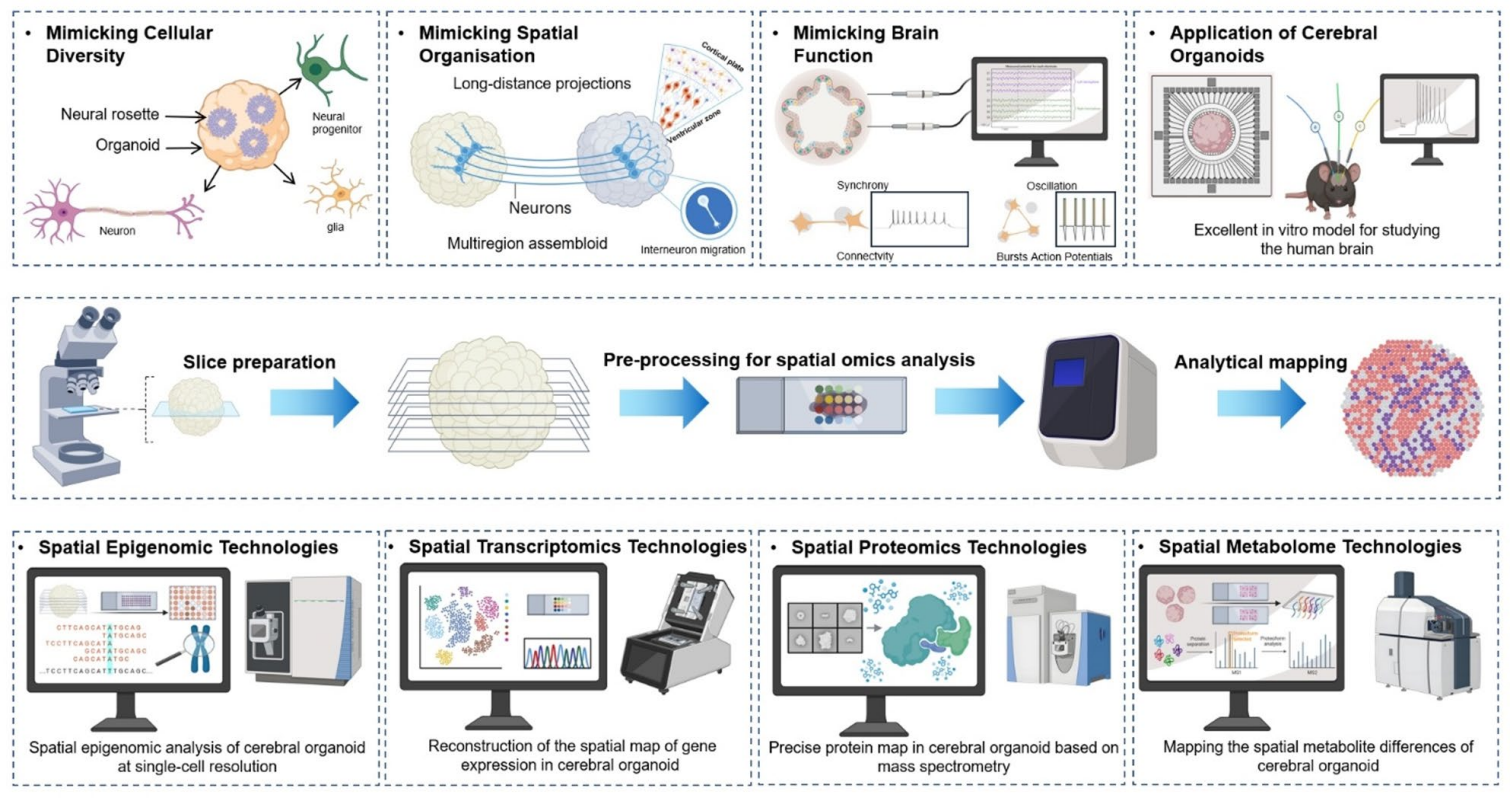
Application of cerebral organoids and spatial omics in studying central nervous system. Cerebral organoids derived from human pluripotent stem cells provide solutions for the cell diversity, spatial structural complexity, and richness of brain functions of the central nervous system. Cerebral organoids have become an ideal platform for studying the physiological development and pathological mechanisms of the human brain. Spatially resolved multi-omics technologies have enabled comprehensive multidimensional profiling of neurophysiological and neuropathological developmental processes in cerebral organoids at single-cell resolution

The convergence of these technologies forms a synergistic platform that directly tackles the core challenges of decoding the brain’s structural complexity, molecular heterogeneity, and high-dimensional dynamics. This approach transcends the limitations of two-dimensional models, propelling brain research into a three-dimensional paradigm that unlocks the ability to explore high-dimensional complexity and precisely investigate the pathophysiology of neurodevelopmental disorders [25, 26]. This review first delineates the transformative contributions of cerebral organoids and spatial multi-omics to developmental neuroscience. We then discuss their combined potential, wherein spatial multi-omics provides profound insights into the spatial architecture and molecular dynamics of organoids [27]. Although this integration remains nascent, we outline conceptual frameworks demonstrating its power to reshape the study of neurogenesis, neuroconnectivity, and neuroregeneration. Finally, we envision future opportunities and challenges, proposing that this synergistic approach can catalyze a new era of research and therapeutic innovation in stem cell-based treatments for pediatric neurodevelopmental disorders (Fig. 1).

## Cerebral organoids: mimicking the human brain

Brain development is a self-organizing process involving cell proliferation, differentiation, migration, and the formation of functional neural circuits [28]. In humans, this complex process unfolds during the fetal period and extends into postnatal stages, making direct research on functional human brain tissue challenging. Cerebral organoids, derived from human pluripotent stem cells (hPSCs), offer a solution by replicating key features of the human brain, including cellular heterogeneity, structural organization, and physiological responses [29–31]. These organoids mimic brain development by recapitulating intrinsic patterning cues, cellular interactions, and region-specific differentiation driven by self-organizing signaling pathways [32]. This makes cerebral organoids a powerful platform for studying human brain development and disease mechanisms (Table 1), bridging the gap between simplified in vitro models and the complexity of the human CNS [33].

**Table 1:** Examples of neurological diseases modelled with cerebral organoids

Disease	Manifestations	Organoid type	Summary	In vitro stage	Ref.
Epileptic encephalopathy	Intractable, aggressive EEG paroxysmal abnormalities and severe neurocognitive deficits	Undirected	Mosaic PCDH19 hCOs showed VZ WT/KO segregation, altered PCDH19/N-Cadherin expression, and deep-layer neurogenesis defects exclusively in mixed WT: KO models.	~ 7 weeks	Niu, W. et al., 2024 [156]
Juvenile neuronal ceroid lipofuscinosis	Rapidly progressing visual abnormalities	Undirected	CLN3Q352X-mutated cerebral organoids modeling neurodevelopmental disorders showed 50% failed development and 50% synaptic alterations, indicating severe brain developmental impairment from this mutation.	66 days	Gomez-Giro et al., 2019 [134]
Sandhoff disease	Accumulation of fats in neurons	Undirected	Sandhoff disease organoids accumulated GM2 gangliosides, showed enlarged size and increased proliferation vs. gene-corrected; transcriptomics revealed impaired development linked to altered neuronal differentiation in early GM2 accumulation.	14 weeks	Allende et al., 2018 [157]
La Crosse encephalitis virus encephalitis	Headache, fever, nausea, or weakness	Undirected	Cerebral organoids modeled La Crosse virus (LACV) neuronal tropism, revealing mature neurons’ heightened apoptosis susceptibility and IFN signaling’s critical role in virus-induced cell death during neurodevelopment.	~ 33 days	Winkler et al., 2019 [158]
Autism (ASD)	Range of mental disorders that affect communication and behaviour	Undirected	Forebrain organoid scRNA-seq revealed genetic background (not ASD) drives cellular heterogeneity; progenitor states (NEUROD6/EMX1, GSX2/DLX1, ZIC1/WNT) determine neuronal fate, diversity from programmed specification, not stochasticity.	60 days	Jourdon A., et al., 2023 [159]
Down Syndrome (DS)	Flattened face, especially the bridge of the nose; almond-shaped eyes that slant up	ChP-like epithelium	DS ChPCOs (iPSC-derived cortical/ChP-like epithelium) recapitulate abnormal corticogenesis, ChP ciliogenesis/polarity defects, and heightened SARS-CoV-2 neuronal infection, rescued to euploid levels via TMPRSS2/furin inhibition, providing a platform for neurotropic virus and therapeutic research.	56 days	Shaker, M. R et al., 2024 [160]
		Ventral forebrain	DS iPSC brain organoids model neurodevelopmental anomalies (cortical thinning, impaired proliferation/neurogenesis); allele-specific DSCAM knockout rescues deficits, demonstrating dosage-sensitive corticogenesis regulation in Down syndrome.	115 days	Tang XY et al., 2021 [161]
Periventricular heterotopia (PH)	Intellectual disability, frequently associated with epilepsy	Cortical spheroids	FAT4/DCHS1 mutant hCOs model PH neuronal hyperactivity with spontaneous spike surges, sodium channel-mediated excitability, synaptic defects, and morphological complexity, reversed by wild-type DCHS1 rescue in multi-omics-validated 3D systems.	~ 9 months	Di Matteo, F. et al., 2025 [162]
Schizophrenia	Mental disorder characterized by abnormal behaviour and speech	Undirected	PCCB knockdown in hFOs disrupted GABAergic synapses via TCA cycle-linked GABA reduction, causing schizophrenia-like hyperactivity and desynchronization, connecting mitochondrial dysfunction to neuropsychiatric circuit defects.	60 days	Zhang W et al., 2023 [163]
		Undirected	Schizophrenia iPSC cerebral organoids exhibit synaptic/neurodevelopmental gene dysregulation (23% GWAS genes altered: 10↑/15↓), mitochondrial dysfunction (reduced ATP/respiration), and diminished neuronal stimulation response, modeling disease pathways via RNA-seq and electrophysiology.	~ 9 months	Kathuria, A. et al., 2020 [164]
Parkinson’s disease (PD)	Neurodegenerative disorder that progressively impairs movement	Midbrain	Novel midbrain organoids generated mature nigral dopaminergic neurons; GBA1-Parkinson’s models showed progressive neurodegeneration, Lewy-like inclusions, and transmissible α-synuclein fibrils propagating synucleinopathy to healthy organoids.	150 days	Frattini E et al., 2024 [165]
		Undirected	GCase deficiency combined with α-syn overexpression triggers detergent-resistant, β-sheet-rich Lewy body-like aggregates (ubiquitin/α-syn core) in hMLOs, including PD/SNCA triplication models, demonstrating GCase dysfunction drives α-syn pathology.	25–35 days	Jo, J. et al., 2021 [166]
		Cortical spheroids	Alpers’ syndrome (POLG mutations) cortical organoids replicate postmortem brain pathology (neuronal loss, mtDNA/CI depletion); neural stem cells show mitochondrial ROS/NADH defects, reversed by nicotinamide riboside, validating iPSC-organoid models for drug discovery in mitochondrial diseases.	90 days	Hong, Y. et al., 2024 [167]
Alzheimer’s disease (AD)	Neurodegenerative disease characterised by dysfunction in memory, thinking, and behaviour	Cortical spheroids	HSV-1-infected brain organoids show elevated tau phosphorylation (p-tau) reduces neuronal death; STING activation enhances/TBK1 inhibition blocks p-tau, implicating cGAS-STING-driven tau-mediated antiviral response in AD.	~ 50 days	Hyde, V. R et al., 2025 [168]
		Undirected	AD cerebral organoids showed Aβ accumulation, increased tau phosphorylation/neurofibrillary tangles, and elevated apoptosis, all ameliorated by β-/γ-secretase inhibitors (BACE1 inhibitor IV/compound E).	~ 6 months	Choe, M. S. et al., 2024 [152]
		Undirected	3D familial AD cerebral organoids (iPSCs with PSEN1/2 mutations) recapitulate AD pathology markers, developmental patterning defects, and premature neuronal differentiation, confirmed through single-cell transcriptomic analysis.	95 days	Vanova, T. et al., 2023 [169]
Multiple sclerosis (MS)	Muscle weakness or spasms	Forebrain	A SOX10-driven human forebrain organoid model with immunocompetent CNS cells recapitulates chronic multiple sclerosis (MS) neurodegeneration, showing CSF-mediated oligodendrocyte loss (50% by day 6) and inflammatory cascades, enabling drug screening against MS progression.	8 weeks	Fagiani, F. et al., 2024 [170]
		Undirected	MS remyelination failure stems from epigenetically silenced oligodendrocytes. ESI1, an epigenetic-silencing inhibitor, restores myelin in iPSC organoids/aged mice via SREBP1/2 nuclear condensate-driven lipid/cholesterol biosynthesis, reversing cognitive decline and enabling CNS axon remyelination.	12 weeks	Liu, X. et al., 2024 [171]
Microcephaly	Birth defects resulting in a smaller than normal head	Undirected	Patient-derived iPSCs and short hairpin RNA in these organoids modelled CDK5RAP2-dependent pathogenesis of microcephaly, which has been difficult to model in mice.	ཞ75 days	Lancaster et al., 2013 [48]
Hypoxic encephalopathy	Weak or absent breathing patterns, low heart rate, acidosis of the blood, poor muscle tone, and weak or absent reflexes	Cortical spheroids	Human 3D brain organoids modeling hypoxia revealed intermediate progenitor defects (linked to cortical expansion) involving unfolded protein response dysregulation during corticogenesis.	ཞ75 days	Pasca et al., 2019 [172]

### Mimicking cellular diversity

A comprehensive understanding of the complex cellular diversity of the human brain is essential for elucidating the physiological mechanisms of neural function establishment during brain development and the pathogenesis of neurological disorders. Historically, this cellular heterogeneity has been modeled using animal systems or simplified two-dimensional (2D) cell cultures. However, these approaches fail to replicate the intricate composition and developmental trajectories of the human brain. Animal models, such as rodents, exhibit vastly different cell ratios and lineage patterns compared to humans, limiting their translational relevance. By contrast, cerebral organoids, derived from human-induced pluripotent stem cells (hiPSCs), offer a groundbreaking platform that overcomes these limitations by faithfully recapitulating the cellular and molecular heterogeneity of the human brain in vitro.

Cerebral organoids’ ability to replicate the diversity of the human brain arises from their self-organization and the intrinsic developmental cues encoded within hiPSCs [34]. These organoids mimic in vivo brain development through stepwise differentiation of neural progenitors into various neuronal and glial populations. This process is directed by spatiotemporal signaling gradients that resemble those found in the developing human brain, enabling the formation of region-specific structures. For instance, dorsal forebrain organoids reliably produce cell types characteristic of the human cerebral cortex, reflecting a fidelity that is unmatched by other model systems. High-throughput studies, such as single-cell RNA sequencing, have validated this capability. For example, one study analyzed over 166,000 cells from 21 organoids, revealing that 95% of the organoids formed consistent cell types and followed developmental trajectories similar to those of the human brain [35]. Furthermore, transcriptional profiling demonstrated that cerebral organoids align closely with the human fetal brain at distinct developmental stages, providing an accurate timeline for studying cellular transitions and maturation processes [36, 37]. As organoids mature in vitro, they exhibit increasingly complex cell populations, including advanced neuronal and glial subtypes, while recapitulating essential processes such as gliogenesis and synaptogenesis [38].

The ability of cerebral organoids to replicate cellular diversity has profound implications for neuroscience research and therapeutic development. By reproducing the broad spectrum of human brain cell types, organoids provide a platform for investigating key developmental processes, such as cortical layering and neuronal subtype differentiation, which are challenging to study in vivo or in non-human systems [39, 40]. For instance, organoids have enabled researchers to explore the formation of regionalized structures and the dynamics of neural connectivity in ways that were previously inaccessible. The cellular heterogeneity of cerebral organoids also makes them a robust tool for studying disease mechanisms. Many neurological disorders, including autism spectrum disorders, schizophrenia, neurodevelopmental disorder and neurodegenerative diseases, are associated with disruptions in specific cell types or interactions [41]. By using patient-derived organoids, researchers can model these disruptions within a human-specific framework, uncovering cell-type-specific vulnerabilities. For example, cerebral organoids have been pivotal in understanding the mechanisms of microcephaly by highlighting critical defects in neuronal proliferation and function [42, 43].

### Mimicking spatial organisation

The structure of the human brain is a cornerstone of its function, with intricate spatial organization enabling processes such as information processing, connectivity, and plasticity. At the forefront of this complexity is the cerebral cortex, which features a laminar architecture of six distinct neuronal layers [44]. These layers, along with spatially organized progenitor zones like the subventricular zone (SVZ), play a critical role in orchestrating neural development and circuit formation [45, 46]. The unique spatial hierarchy and interconnectivity of these regions are essential for understanding how the brain operates under normal conditions and how disruptions lead to disease [47].

Cerebral organoids, derived from human pluripotent stem cells (hPSCs), represent a cutting-edge tool for studying the intricate architecture of the human brain. The development of cerebral organoids was first pioneered by Lancaster and colleagues, who demonstrated the ability of these structures to recapitulate aspects of children brain development in vitro [48]. Organoids are cultured in three-dimensional (3D) matrices, such as Matrigel, which support the self-organization of cells into layered structures resembling those found in the brain [49]. These layers include progenitor zones (ventricular, subventricular, and intermediate) and neuronal zones (deep-layer neurons, upper-layer neurons, and Cajal-Retzius cells), mimicking the laminar organization of the human cerebral cortex. The structural complexity of cerebral organoids is particularly evident in their ability to replicate the subventricular zone (SVZ), which is far more elaborate than in rodent models. The SVZ in organoids is divided into the inner SVZ (iSVZ) and outer SVZ (oSVZ), separated by the inner fiber layer (IFL), mirroring the architecture of the developing brain. Advanced imaging techniques and single-cell transcriptomics have confirmed that cerebral organoids align closely with the transcriptional profiles and developmental trajectories of the human fetal brain [50].

The ability to fuse region-specific cerebral organoids into multi-region assembloids has further advanced the modeling of brain architecture [51, 52]. This technique enables researchers to simulate interregional interactions that occur during brain development, such as the migration of interneurons from the ventral forebrain to the dorsal forebrain. For example, ventral forebrain organoids, which generate gamma-aminobutyric acid (GABA)-ergic interneurons, have been fused with dorsal forebrain organoids to model interneuron migration. These assembloids mimic the processes by which cortical interneurons integrate into cortical circuits, offering insights into developmental mechanisms that are disrupted in conditions such as epilepsy and ASD. Notable studies have highlighted the utility of assembloids for structural and functional modeling. Xiang et al. fused medial ganglionic eminence (MGE) organoids with cortical organoids to replicate interneuron migration and integration into cortical circuits [53, 54]. This model closely resembles the ventral-to-dorsal migration observed during brain development and has provided valuable insights into the molecular mechanisms underlying interneuron migration. Similarly, Birey and colleagues created forebrain assembloids to study neuronal migration in the context of Timothy syndrome, a neurodevelopmental disorder [55]. Their findings demonstrated abnormal migratory behavior of interneurons in patient-derived assembloids, emphasizing the potential of this system for disease modeling.

### Mimicking brain function

Cerebral organoids have emerged as powerful models for replicating the intricate functionality of the human brain. Beyond their ability to mimic cellular diversity and structural complexity, these organoids demonstrate neural network activity, dynamic axonal behaviors, and the capacity to integrate into host neural circuits [56–58]. These capabilities provide a comprehensive platform for exploring the functional principles of brain development, connectivity, and behavior.

A defining aspect of brain functionality is the presence of neural oscillations, rhythmic electrical signals that govern communication and coordination within neural circuits. Cerebral organoids have shown remarkable ability to recapitulate these oscillatory dynamics. Over a five-month period, multi-electrode array studies revealed hallmark features of neural circuit maturation in organoids, such as rapid firing rates and synchronized network-bursting events. These events, driven by glutamatergic and GABAergic signaling, highlight the physiological relevance of organoid-derived neural activity [59, 60]. To contextualize these findings in human neurodevelopment, a machine learning model was used to compare electroencephalogram (EEG) features from cerebral organoids and human preterm infants. The model successfully aligned the developmental trajectories of organoids with those of infants, reinforcing their ability to replicate early neurodevelopmental dynamics. These results demonstrate that cerebral organoids can faithfully mimic not only structural and molecular aspects of the brain but also its dynamic functional processes [61].

In addition to network-level dynamics, cerebral organoids exhibit advanced axonal behaviors that mirror key features of in vivo neural connectivity. These include axon guidance, long-range projection formation, growth cone turning, and decussation. Such behaviors underscore the ability of organoids to simulate the physical and functional properties of developing neural circuits. One striking example of this functionality comes from studies co-culturing cerebral organoids with mouse spinal cord-muscle explants [62]. In this setup, axonal projections from the organoids induced coordinated muscle contractions in the mouse tissue, providing direct evidence of functional neural output. Further advancing this approach, cortico-motor assembloids—created by fusing cortical, hindbrain, and spinal cord organoids with skeletal muscle spheroids—recapitulated motor circuits capable of triggering robust muscle contractions upon optogenetic stimulation of cortical regions [33]. These assembloids maintained functionality for over 10 weeks, offering a long-term platform to study motor control and its developmental disruptions.

Cerebral organoids extend their functional capabilities through integration into host neural environments, enabling researchers to investigate human brain functionality in vivo [63]. When transplanted into rodent brains, organoids demonstrated enhanced maturation, forming synaptic connections, extending axons, and receiving inputs from host thalamocortical and corticocortical circuits [64, 65]. This integration fostered advanced morphological and synaptic properties that surpassed those observed in vitro, emphasizing the role of the in vivo environment in promoting organoid development. Remarkably, these transplanted organoids influenced host behavior. For instance, optogenetic activation of organoids transplanted into the somatosensory cortex of rodents evoked sensory responses and reward-seeking behaviors [66]. This functional integration highlights the ability of cerebral organoids to engage with host neural networks, providing an unprecedented opportunity to study neural development processes and their behavioral implications in living systems.

## Spatial multi-omics: illuminating the brain research

The spatial multi-omics approach is transforming our understanding of the brain by providing detailed insights into the spatial organization and molecular interactions within neural tissues. By integrating genome, transcriptome, proteome, metabolome, and epigenome data in their spatial context, this methodology enables researchers to explore how diverse molecular processes converge to regulate brain development and function [67, 68]. With its ability to map cellular and molecular interactions while preserving the brain’s complex architecture, spatial multi-omics offers a powerful tool for uncovering the mechanisms underlying neural development, differentiation, synaptic connectivity, and region-specific specializations [69–71]. In the foreseeable future, this transformative approach is expected to comprehensively elucidate the molecular mechanisms governing childhood brain development and the pathogenesis of neurological disorders from a three-dimensional spatial perspective, thereby advancing both fundamental neurodevelopmental research and the development of targeted therapeutic interventions.

### Spatial epigenomic technologies

Spatial epigenomic technologies, first introduced to resolve the spatial complexities of epigenetic regulation, have become indispensable tools in neuroscience. These methods enable the precise mapping of epigenetic modifications, such as histone modifications, DNA methylation, and chromatin accessibility, within their spatial context in tissues [72, 73]. By preserving tissue architecture, these approaches allow researchers to uncover intricate relationships between spatial epigenetic patterns and cellular states, offering transformative insights into brain development, function, and disease mechanisms.

A significant advancement in the field is epigenomic MERFISH, developed by Zhuang and colleagues [74]. This technique combines CUT&Tag (cleavage under targets and tagmentation) with multiplexed error-robust fluorescence in situ hybridization (MERFISH), enabling spatially resolved epigenomic profiling at single-cell resolution [75]. By simultaneously mapping the spatial distribution of over 100 epigenomic loci in tissues, epigenomic MERFISH focuses on histone modifications associated with active promoters, enhancers, and silent chromatin. Applied to brain tissue, this method has revealed enhancer hubs and promoter–enhancer interactions critical for neuronal development and brain region specification. These spatially detailed insights underscore the utility of epigenomic MERFISH in dissecting the epigenetic architecture underlying brain arealization and the establishment of functional networks.

The key innovation is spatial-CUT&Tag, introduced by Fan et al. [73]. This genome-wide profiling technique combines in situ CUT&Tag chemistry with microfluidic deterministic barcoding and next-generation sequencing (NGS). While spatial-CUT&Tag provides lower spatial resolution compared to epigenomic MERFISH (approximately 100-fold lower), it excels in unbiased, genome-wide mapping of epigenetic modifications. Studies on mouse and human brain tissues using this approach have identified histone modification patterns linked to neuronal differentiation, glial specification, and regional brain identity, offering a comprehensive view of the epigenetic mechanisms driving brain development [76, 77].

Complementing these methods is spatial-ATAC-seq, which profiles chromatin accessibility in tissue sections. By employing in situ Tn5 transposition chemistry combined with microfluidic barcoding, spatial-ATAC-seq maps accessible genomic regions, providing insights into the spatial organization of regulatory elements. In mouse and human brain studies, this method has delineated chromatin accessibility patterns across distinct brain regions, revealing how regulatory landscapes shape neuronal and glial lineage commitment during development [72]. Spatial-ATAC-seq has also proven valuable for identifying active enhancers and super-enhancers driving region-specific gene expression in the brain. Today, Spatial-ATAC has emerged as a groundbreaking technology in the field of epigenomics. It leverages sequencing to perform spatial analysis of transposase-accessible chromatin, enabling the examination of chromatin accessibility in intact tissue sections at nearly single-cell resolution. Spatial-ATAC has already been applied to study tissue-region-specific epigenetics in mouse embryos and to identify key gene regulators involved in the development of the central nervous system [78].

Another innovative advancement in epigenomics research is MISAR-seq, a technology that integrates spatial epigenomics and transcriptome co-analysis. This approach allows for the simultaneous mapping of chromatin accessibility and mRNA expression within the same tissue section. Currently, MISAR-seq has been utilized to investigate the development of the mouse brain across different embryonic stages, offering new insights into the dynamic environmental regulation of brain development [79]. By combining the perspectives of the epigenome and transcriptome, this technology provides a novel methodological framework for biomedical research, deepening our understanding of the interplay between genes and chromatin structure at the spatial level [80].

### Spatial transcriptomics technologies

In 2016, Ståhl and colleagues introduced a next-generation spatial transcriptomics method based on barcoded arrays, marking a significant milestone in the field [81]. This technique utilizes spatially resolved barcodes to capture RNA transcripts while preserving their positional information within tissue slices. Tissue sections are placed on slides embedded with spatially encoded oligonucleotides, enabling the capture and subsequent sequencing of RNA from specific regions. Each spot on the array contains unique barcodes corresponding to its spatial coordinates, allowing for the reconstruction of gene expression maps across the tissue. One of the earliest applications of this method was in the study of the mouse olfactory bulb, where over 1,000 spatial spots were analyzed to provide an unbiased, transcriptome-wide view of gene expression patterns [82, 83].

Since its introduction, the technique has evolved through innovations such as Slide-seq and Slide-seqV2, which employ dense bead arrays to achieve near single-cell spatial resolution [84]. These methods enable high-throughput mapping of gene expression across complex tissues, such as the cerebral cortex, revealing intricate patterns of neuronal and glial gene expression. Despite their power, these next-generation sequencing (NGS)-based techniques rely on tissue sectioning and offer limited subcellular resolution compared to imaging-based approaches.

In contrast, imaging-based spatial transcriptomics methods, such as in situ sequencing (ISS) and in situ hybridization (ISH), directly visualize RNA molecules within their tissue context [85, 86]. ISS involves reverse transcription of RNA followed by rolling circle amplification and sequencing within the tissue, enabling high-resolution spatial transcriptomic profiling. ISH techniques, such as multiplexed error-robust fluorescence in situ hybridization (MERFISH), use fluorescent probes to target specific RNA sequences, allowing researchers to detect thousands of transcripts simultaneously. These imaging methods have proven particularly valuable for studying the spatial organization of gene expression within neural circuits, where fine resolution is essential for correlating molecular data with neuronal morphology and connectivity [87, 88].

One transformative application of these technologies in neuroscience is the construction of spatially resolved transcriptomic atlases of the brain. By integrating spatial transcriptomics with anatomical mapping, researchers have classified neural cell types across brain regions based on their unique gene expression profiles. For example, the adult mouse brain was comprehensively profiled using spatial transcriptomics, capturing over 15,000 genes across 34,000 spatial points [89]. This effort provided a molecular map linking gene expression to specific anatomical and functional brain regions, offering a framework for understanding the relationship between brain structure and function. Spatial transcriptomics has also been applied to study neural circuits in the cerebral cortex, uncovering the molecular diversity underlying long-range projections. A notable example is the use of Barseq2, a high-throughput spatial transcriptomics method, which maps gene expression alongside neuronal projections to multiple brain regions [90]. This approach revealed previously unrecognized cadherin family members associated with homologous projections between cortical areas, shedding light on the molecular mechanisms governing neural connectivity.

Stereo-seq, an advanced spatial transcriptomics technology developed by BGI, represents a leap forward in the field [91]. Its key features include unparalleled ultra-high resolution and an ultra-large field of view. By utilizing DNA nanoball (DNB) patterned arrays, Stereo-seq achieves 500-nanometer resolution and captures a greater number of genes, enabling spatial positioning and identification of molecular information at the single-cell level [92]. This technology has made significant strides in practical applications, achieving breakthroughs in various feasibility tests. For instance, Science Plus employed Stereo-seq to perform high-resolution spatial transcriptome analysis on 62 CS8 human embryo slices through continuous sectioning, subsequently reconstructing a 3D model of the human CS8 embryo [93, 94]. Additionally, the world’s first multi-organ aging atlas based on Stereo-seq technology systematically mapped differences in immunoglobulin G (IgG) during aging, revealing immunoglobulin aging as a hallmark of aging [95]. Further breakthroughs have been achieved through the integration of Stereo-seq with organoid-based technology (LOSRT), enabling the construction of single-cell resolution spatial transcriptome maps of mouse liver and lung organoids [96, 97]. Similarly, DNA array-based spatial transcriptomics sequencing has facilitated applications ranging from fundamental developmental studies of tissues and embryos to its integration with cerebral organoids. Lozachmeur G et al. established a low-cost strategy for producing dual-barcode DNA arrays. Applying this technology, they achieved multiplex gene profiling in human brain organoids, thereby delineating their heterogeneous gene expression atlas [98].

These studies have unveiled interaction and communication patterns between different cell types within organoids, opening new possibilities for future drug development models. Stereo-seq technology provides unprecedented resolution of molecular dynamics within tissues, pushing spatial transcriptomics to new heights. With the advent of higher-resolution spatial multi-omics technologies, humanity is poised to gain a deeper understanding of the molecular mechanisms underlying life activities [99, 100].

### Spatial proteomics technologies

In recent years, spatially resolved proteomics has emerged as a transformative approach for investigating the spatial organization of the proteome within tissues, particularly in the brain. This technique allows for the precise mapping of protein abundance, localization, and post-translational modifications (PTMs), providing critical insights into cellular heterogeneity and molecular interactions within neural circuits. Spatial proteomics methodologies can be broadly categorized into imaging-based approaches and mass spectrometry-based techniques, each offering unique advantages for analyzing brain tissue [101].

Imaging-based methods, such as immunohistochemistry (IHC), are foundational to spatial proteomics [102]. IHC uses antibodies to target specific proteins within brain tissue sections, visualized through chromogenic or fluorescent labeling. This approach enables high-resolution mapping of protein localization within the brain’s structural context, revealing spatial patterns of expression in neurons, glial cells, and synaptic regions. Advanced multiplex IHC further enhances this technique by detecting multiple proteins simultaneously, providing detailed insights into protein co-expression and interactions in neural networks. Recent innovations, including automated staining systems and improved antibody specificity, have increased the throughput and reproducibility of IHC, making it an indispensable tool for studying protein distributions in the brain [103, 104].

Mass spectrometry (MS)-based approaches offer a complementary perspective by quantifying proteins and their modifications across spatially defined brain regions. Early implementations of spatial proteomics using MS involved manual dissection of brain sections into micro-regions, but these methods faced limitations in spatial resolution and sensitivity. To overcome these challenges, technologies such as laser microdissection (LMD) coupled with advanced sample preparation techniques like NanoPOTS (nanodroplet processing in one pot for trace samples) have been developed [105–107]. These methods allow high-precision proteomic analysis of subregions in the brain, such as the hippocampus or prefrontal cortex, and have been instrumental in uncovering protein networks involved in synaptic plasticity and memory formation.

One significant advancement in spatial proteomics is the ProteomEx method, which combines tissue amplification with mass spectrometry-based proteomics [108]. ProteomEx achieves lateral resolutions of approximately 160 μm, enabling detailed mapping of neurotransmitter-related proteins and synaptic markers in the brain. This technique has been particularly valuable for studying regional protein variability in neurodegenerative diseases, such as Alzheimer’s and Parkinson’s, providing insights into early molecular changes associated with disease progression [109, 110].

To address the challenges of comprehensive spatial analysis across entire brain sections, microstent-assisted spatial proteomics (MASP) was developed [111]. MASP employs 3D-printed microstents to compartmentalize brain tissue, enabling the analysis of thousands of proteins with high quantitative accuracy. This method is particularly well-suited for investigating region-specific pathways, such as those involved in synaptic signaling or neuroinflammation, and has been applied to map neurotransmitter transporters and drug targets in the brain. MASP provides a deeper understanding of the spatial dynamics of protein expression in the brain, complementing traditional imaging and MS-based approaches. Another breakthrough in spatial proteomics is the DISCO-MS platform, which integrates panoramic imaging and AI-driven 3D tissue reconstruction to achieve unbiased proteomic analysis of brain regions [112].

The spatial CITE-seq technology platform represents a significant advancement, enabling the in situ co-detection of the entire transcriptome and multiple proteins at single-cell resolution within the same tissue slice. This capability has greatly enhanced our understanding of the intricate relationships between genes and protein interactions. By leveraging this technology, researchers can now observe spatial changes in protein states and gene activity during embryonic development, providing deeper insights into the key roles and mechanisms of genes and proteins in the evolution of life [113, 114].

Another groundbreaking innovation is STARmap PLUS, a cutting-edge spatial transcriptomics technology that integrates high-throughput gene expression analysis with proteomics at three-dimensional spatial resolution. This method combines molecular barcoding, hydrogel embedding, in situ cDNA synthesis, rolling circle amplification (RCA), sequential fluorescence hybridization, three-dimensional spatial mapping, and high-throughput computational analysis to achieve comprehensive spatial gene expression mapping and single-cell RNA sequencing (scRNA-seq) [115]. STARmap PLUS has emerged as a revolutionary tool for studying spatial gene expression patterns in the brain, offering unprecedented resolution and detail [116].

### Spatial metabolome technologies

In recent years, spatial metabolomics has emerged as a critical technology for understanding the spatial organization of metabolic processes within tissues, particularly in the brain [117, 118]. This approach allows researchers to map metabolites, lipids, and small molecules in their native spatial contexts, providing unparalleled insights into the metabolic mechanisms that underlie brain function, connectivity, and pathology. By correlating metabolic alterations with their precise locations, spatial metabolomics has advanced our understanding of brain region specialization, disease progression, and the search for novel biomarkers.

The core technology behind spatial metabolomics is imaging mass spectrometry (imaging MS), which integrates spatially resolved molecular sampling with mass spectrometric detection [119]. Imaging MS enables the simultaneous analysis of hundreds of metabolites, offering a molecular atlas of brain regions with high spatial precision. The technique is broadly categorized based on ionization sources, such as matrix-assisted laser desorption/ionization (MALDI), desorption electrospray ionization (DESI), and secondary ion mass spectrometry (SIMS), each with unique strengths for brain research [120–122].

In MALDI-based techniques, laser-induced ionization is used to ablate molecules from tissue sections, enabling spatially resolved analysis of metabolites. This approach allows the precise localization of metabolic alterations in specific brain regions. For instance, Khalil SM established a methodology for high-resolution MALDI-MSI of human brain organoids, enabling the precise mapping of metabolites such as amino acids and thereby providing deeper insights into brain development and cellular metabolic fate trajectories [123]. These findings highlight the potential of MALDI-based methods for uncovering regional vulnerabilities in neurodegenerative diseases. Similarly, DESI-based methods utilize liquid ionization to detect metabolites directly from tissue surfaces, preserving the chemical integrity of delicate molecules such as neurotransmitters and small lipids [124]. This technique has proven particularly effective in studying glial cell metabolism and its interactions with neurons in conditions such as multiple sclerosis. By mapping lipid distributions in the hippocampus and cortex, DESI imaging has provided critical insights into neuroinflammatory processes and their impact on cognitive decline. For subcellular-level analysis, SIMS-based techniques use an ion beam to achieve nanometer-scale resolution, making them ideal for studying synaptic metabolism. SIMS imaging has been instrumental in visualizing the spatial dynamics of neurotransmitters, such as glutamate and GABA, within individual synapses. This has shed light on synaptic dysfunction in epilepsy and depression, linking metabolic imbalances to disrupted neurotransmitter cycling.

### Spatial protein and transcriptome sequencing technologies

Spatial protein and transcriptome sequencing (SPOTS) combines transcriptomics with spatial protein analysis, providing comprehensive insights into cellular heterogeneity, tissue structure, and molecular interactions within biological systems [125]. One of the key breakthroughs in this field is the development of advanced computational methods to improve the accuracy of spatial transcriptomics. For example, an imputation method using graph-regularized tensor completion has been proposed to address the challenges of data sparsity and missing values, enabling the reconstruction of spatially resolved transcriptomes with greater precision [126]. Additionally, Redeconve, a computational tool for deconvolving spatial transcriptomics data at single-cell resolution, has proven effective in accurately mapping cell-specific gene expression [127].

The application of SPOTS in physiological and pathological studies has demonstrated its potential to uncover the mechanisms underlying various biological processes. For instance, a study using spatial transcriptomics to analyze hepatitis B virus (HBV) integration in patients with HBsAg loss revealed that the level of transcriptionally active viral integration was lower than previously expected [128]. In cancer research, spatial transcriptomics has been applied to study digestive system tumors, showcasing how the integration of transcriptome sequencing and protein markers can distinguish tumor subpopulations and reveal their interactions with the surrounding matrix and immune cells [129].

Moreover, a separate study leveraging integrated spatial transcriptomics and proteomics analyzed multiple cortical and subcortical regions in postmortem brains of COVID-19 patients, revealing consistent dysregulation of neuronal mitochondria and synapses alongside upregulated glial inflammatory responses at both mRNA and protein levels [130]. In parallel, spatial transcriptomics has been successfully applied to map neural circuits and elucidate molecular mechanisms underlying neurological disorders such as Alzheimer’s and Parkinson’s disease. These efforts demonstrate how spatially resolved multiomics can unveil previously unrecognized cellular functions in brain tissue [131, 132]. Looking forward, the combined use of spatial multi-omics with cerebral organoid models holds great potential to systematically decode drug effects and mechanisms in neurodevelopmental disorders, thereby accelerating mechanistic discovery, therapeutic development, and ultimately improving clinical outcomes in pediatric brain diseases.

## The prospect of spatial multi-omics meeting cerebral organoids

**Fig. 2:**
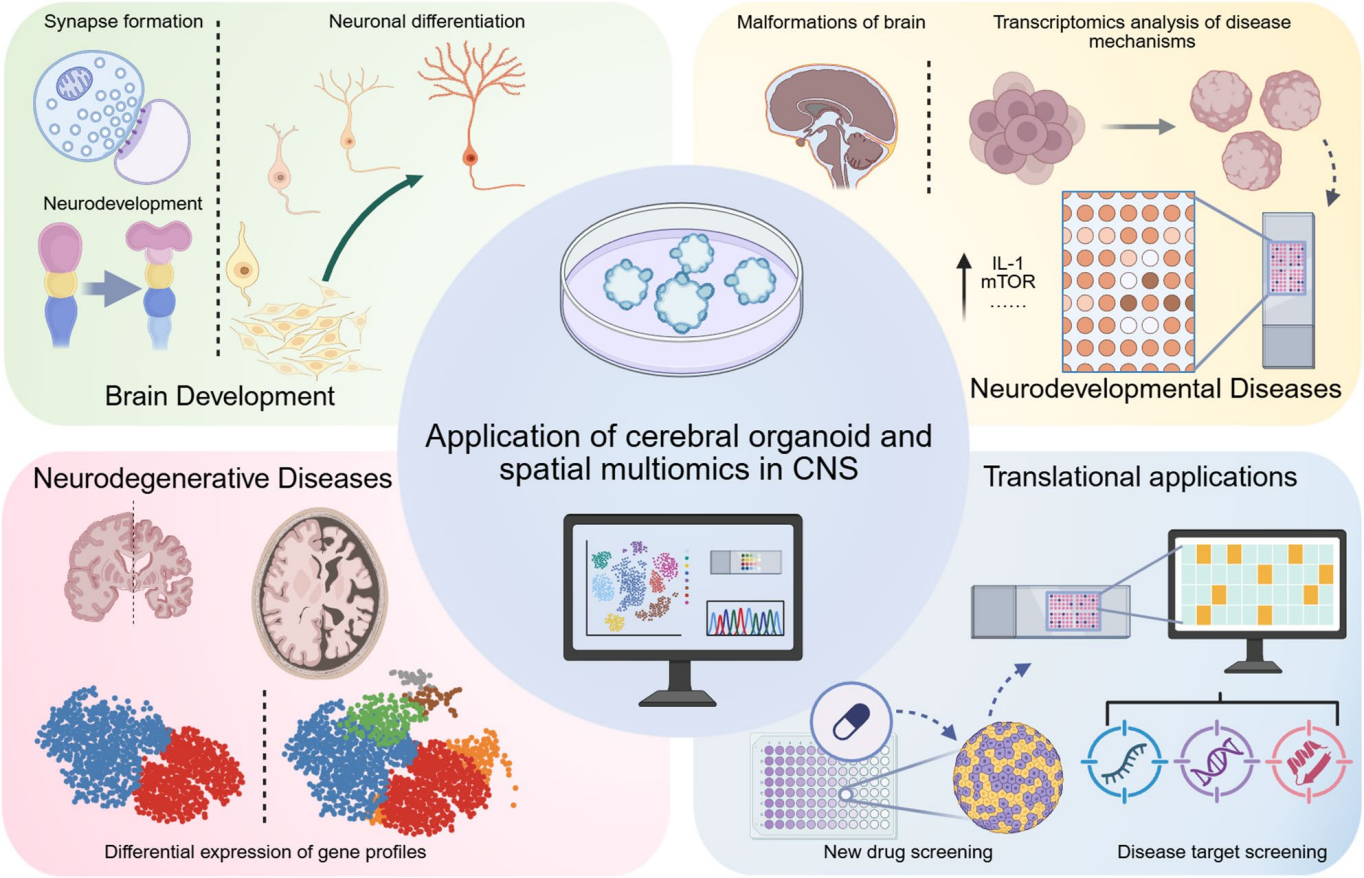
Prospects and potential of spatial multi-Omics combined with cerebral organoids. The integration of spatial multi-omics technologies with cerebral organoid models has propelled brain science research into a new era. From elucidating physiological brain development to unraveling degenerative pathologies and developmental disorders, this synergistic approach is revolutionizing our understanding of neurological systems. These advancements not only illuminate molecular and cellular interactions with unprecedented precision but also open transformative avenues for decoding disease mechanisms and accelerating therapeutic discovery

The integration of spatial omics technologies with cerebral organoids represents a transformative advance in neuroscience, enabling unprecedented insights into the cellular, molecular, and spatial organization of the human brain. Cerebral organoids, as three-dimensional, human-specific brain models, faithfully recapitulate critical aspects of brain development, including morphology, gene expression, and protein dynamics. Combined with spatial omics—such as spatial transcriptomics, proteomics, metabolomics, and epigenomics—these models allow researchers to map gene gradients, protein networks, metabolic pathways, and regulatory elements within their native spatial context. This powerful synergy has revealed intricate processes like cortical layer development, synaptic connectivity, and regional differentiation while uncovering molecular mechanisms underlying brain disorders [133] (Fig. 2).

## Brain development

The integration of cerebral organoids with spatial multi-omics technologies holds great potential to transform our understanding of brain development and function. Cerebral organoids, which mimic the structural organization and regional characteristics of the human brain, provide a powerful in vitro model for studying morphogenesis, cell differentiation, and neural connectivity. When combined with spatial multi-omics tools, this system enables unprecedented insights into brain processes at both spatial and molecular levels [69].

For instance, spatial transcriptomics can map gene expression gradients within organoids, revealing molecular cues that drive cortical layer formation and neuronal subtype differentiation. Similarly, spatial proteomics offers a means to visualize protein localization and dynamics, shedding light on processes such as synaptogenesis and neuronal signaling. Spatial metabolomics can uncover the metabolic landscapes that support neural development, including the distribution of neurotransmitter precursors and energy substrates critical for synaptic activity [134]. Moreover, spatial epigenomics allows researchers to map regulatory elements and chromatin modifications that influence neural lineage specification and regional identity.

By combining these advanced technologies, cerebral organoids could serve as a bridge to better understand the interplay between spatial organization and molecular mechanisms in brain development. While this potential is still being realized, the synergy between cerebral organoids and spatial multi-omics provides a promising framework for uncovering the complexities of neural architecture, offering critical insights into brain disorders and paving the way for innovative therapeutic strategies.

## Neurodevelopmental diseases

Defects in neural stem cell proliferation, neuronal differentiation, and synapse formation are central to neurodevelopmental diseases, disrupting the complex processes that shape the developing brain. While traditional models have provided limited insights, cerebral organoids, which closely mimic the structural and functional features of the early fetal brain, have emerged as transformative tools for studying these disorders. Organoid-based models have successfully recapitulated conditions such as microcephaly, lissencephaly, ASD, and Rett syndrome, offering unique opportunities to explore the cellular and molecular mechanisms underlying disease pathology [3, 48, 135, 136]. For instance, cerebral organoids have been instrumental in modeling developmental deficits, such as cortical folding defects in lissencephalies, ZIKV-induced microcephaly, and synaptic dysfunctions in Rett syndrome. These systems provide a window into how neurodevelopmental processes are altered during fetal brain formation, enabling researchers to trace the origins of these disorders.

The integration of spatial multi-omics technologies with cerebral organoids represents a significant advance, offering unprecedented spatial resolution to uncover disease-specific molecular patterns within human-relevant models. While still in its infancy, this approach has the potential to map gene expression, protein distribution, and metabolic activity across organoid structures, preserving the spatial context critical to understanding disease progression. For example, spatial transcriptomics applied to focal cortical dysplasia type IIb (FCD IIb) identified enrichment of pathways such as mTOR signaling, autophagy, and inflammatory responses in distinct lesion regions pathology [137]. These findings highlight the utility of spatially resolved gene expression analysis in revealing region-specific dysfunctions that contribute to neurodevelopmental disease pathology. Similarly, spatial transcriptomic tools like the 10x Genomics Visium platform have been used to map gene expression across the layered architecture of the human cortex. This approach revealed that genes associated with neurodevelopmental disorders, such as ASD and schizophrenia, show preferential expression in specific cortical layers, providing insights into disease susceptibility tied to spatial organization [138]. When applied to cerebral organoid models, spatial transcriptomics can identify disease-specific brain regions and cellular populations affected by genetic mutations or environmental insults. For instance, integrating spatial omics into models of ASD or Rett syndrome could reveal regional disruptions in gene expression that drive altered synaptic development and connectivity, bridging spatial architecture with molecular dysfunction. Similarly, spatial metabolomics holds the potential to map metabolic shifts within organoids, offering insights into how energy imbalances and neurotransmitter metabolism contribute to developmental defects.

## Neurodegenerative diseases

Neurodegenerative diseases (NDDs), such as Alzheimer’s disease (AD), Parkinson’s disease (PD), and frontotemporal dementia (FTD), as well as early-onset neurodegenerative diseases in children, are characterized by progressive neuronal degeneration, malfunction, and loss of neural cell function (Table 2). These conditions arise from a combination of factors, including toxicity, protein aggregation, ageing, and genetic mutations, and are marked by long asymptomatic prodromal periods followed by progressive, age-related pathology. A defining feature of many NDDs is the accumulation of abnormal protein aggregates, such as amyloid-β, tau, and α-synuclein, yet the underlying mechanisms driving their formation and propagation remain poorly understood.

**Table 2:** Examples of the application of Spatial multi-omics in central nervous system diseases

Disease	Spatial omics	Method	Sample	Highlights	Ref.
Alzheimer’s disease (AD)	• Spatial transcriptomics	• In Situ Sequencing	Human and Mouse Brain Tissues	The study identified a network of amyloid-plaque-induced genes (PIGs) involving 57 genes, primarily in microglia and astrocytes located at the periphery of amyloid plaques.	Chen WT et al., 2020 [142]
Alzheimer’s disease (AD)	• Spatial proteomics	• Mass Spectrometry integrated with Laser Capture Microdissection	Human hippocampal brain tissues	The study employed spatial proteomics to dissect the hippocampal subfield-specific pathology associated with Alzheimer’s disease and primary age-related tauopathy.	Walker JM et al., 2024 [173]
Alzheimer’s disease (AD)	• Spatial metabolomics	• Air Flow-Assisted Desorption Electrospray Ionization Mass Spectrometry Imaging	Mouse Brain Tissue (AD Model)	Comprehensive Metabolic Mapping: The study used AFADESI-MSI to map metabolic changes across different brain regions in an Alzheimer’s disease mouse model.	Fan et al., 2024 [174]
Alzheimer’s disease (AD)	• Spatial proteomics • Lipid Imaging	•Matrix-Assisted Laser Desorption/Ionization-Based Mass Spectrometry Imaging •Liquid Chromatography-Tandem Mass Spectrometry •Laser Microdissection	Postmortem Human Brain Tissues	MALDI-MSI visualized Aβ proteoforms (full-length/truncated) in AD brains, revealing aggregation-linked MAPs (MAP1A/B/2) and AD-associated proteins (APP, APOE) via integrated LC-MS/MS analysis.	Toyama et al., 2024 [175]
Traumatic brain injury (TBI)	• Spatial proteomics	• Laser Microdissection • Label-free Quantitative Proteomics	Mouse Brain Tissue (TBI Model)	TBI disrupted glucose/lipid metabolism and activated cholesterol synthesis in dentate gyrus regions; stratum moleculare showed elevated FN1/LGALS3BP, indicating immune involvement post-injury.	Maity S et al., 2024 [176]
Traumatic brain injury (TBI)	• Spatial multi-omics	• Spatial transcriptome and Spatial metabolism	Human Brain Samples	Spatially consistent genes/metabolites identified as biomarkers for brain injury metabolic changes (lipid peroxidation, myo-inositol imbalance) across analytical methods.	Zheng P et al., 2023 [177]
Parkinson’s disease (PD)	• Spatial transcriptomics	• 10x Genomics Visium • Single Nucleus RNA Sequencing • T-cell Receptor Sequencing	Post-mortem Brain Tissue	Study uncovers T-cell spatial reorganization and multicellular networks with astrocytes/myeloid cells in diseased brains, alongside region-specific astrocyte activation driving heterogeneous glial pathology.	Jakubiak et al., 2024 [178]
Multiple sclerosis (MS)	• Spatial transcriptomics	• Single-Cell Transcriptomics and Proteomics • Spatial RNA Sequencing	Human Samples	Progressive MS brains harbor pathogenic CD161+/LTB + T cells; CNS colonization may initiate early, evidenced by natalizumab-mobilized bloodstream presence in relapsing-remitting patients.	Kaufmann M et al., 2021 [179]
Multiple sclerosis (MS)	• Spatial metabolomics	• Matrix-Assisted Laser Desorption/Ionization Mass Spectrometry Imaging	Mouse Brain Tissues	Teriflunomide disrupts purine/pyrimidine and glutathione/carbohydrate metabolism, crosses BBB to alter CNS metabolic pathways.	Rzagalinski et al., 2019 [180]
Amyotrophic lateral sclerosis (ALS)	• Spatial transcriptomics	• Spatial Transcriptomics • Base Scope Validation	Human Post-Mortem Tissues	Spatial transcriptomics in ALS identified 16 dysregulated genes (GRM3/USP47) driving pathways, linking regional vulnerability to therapy via multi-omics integration.	Gregory JM et al., 2020 [181]
Amyotrophic lateral sclerosis (ALS)	• Spatial transcriptomics	• Spatial Transcriptomics Technique • Hierarchical Generative Probabilistic Model	Human Tissues	Mapped transcriptomic changes in motor neurons and glial cells in ALS, identifying key pathways involved in neurodegeneration.	Maniatis et al., 2019 [182]
Amyotrophic lateral sclerosis (ALS)	• spatial transcriptomics	• Spatial E	Human motor cortex tissues	Spatial analysis revealed 260 ALS-associated genes enriched in upper motor neuron-rich motor cortex layer 5; NOMO1 identified via LoF variant burden analysis.	Guo J et al., 2024 [183]
focal cortical dysplasia type II	• Spatial multi-omics	• Mass Spectrometry Imaging • Laser Capture Microdissection • Bulk Transcriptomics	Mouse Model: FCD type II (Rheb); Human cortex	This research provides a comprehensive view of how somatic mutations in FCD type II impact brain function, ing potential therapeutic targets based on proteomic and transcriptomic analyses.	Vermeulen I et al., 2024 [184]
Huntington’s disease (HD)	• Spatial proteomics	Matrix-Assisted Laser Desorption/Ionization Mass Spectrometry Imaging	Mouse Model: YAC128 model	HD mouse model study identified 22 differentially expressed proteins linked to neuronal loss, validated by GFAP/NeuN immunohistochemical staining.	Karayel-Basar et al., 2022 [185]
Stroke	• Spatial transcriptomics • Spatial proteomics	•Tissue-Digital Microfluidic Isolation of Single Cells for -Omics • Visium Spatial Transcriptomics • 10X Chromium Single-Cell Transcriptomics	Mouse brain tissue (post-ischemic stroke)	Integrated Visium/10X spatial transcriptomics revealed injury-proximal astrocyte zonation with perilesional lipid shuttling (Apoe/Fabp5), resolving stratified repair mechanisms and validating tDISCO’s glial spatial dynamics interrogation.	Scott EY et al., 2024 [186]
Stroke	• Spatial proteomics	• Digital Spatial Profiling	Mouse Model: MCAO	Stroke spatial proteomics show core-penumbra zonation: lesion Iba1/CD45/p-tau, peri-infarct BAG3/CTSD, highlighting penumbral proteostasis targets vs. intact PiNT.	Noll JM et al., 2022 [187]
Stroke	• Spatial metabolomics	• LC-QQQ-MS and MALDI-MSI	Rat Model: MCAO	MALDI-MSI metabolomics revealed GHI rescued ischemia-reperfusion injury by restoring 12/23 energy metabolites via glycolytic/TCA/nucleic remodeling, correlating with infarct reduction and neuronal salvage across brain circuits.	Wang H et al., 2023 [188]
Stroke	• Spatial transcriptomics	• Single-cell RNA Sequencing	Mouse brain tissues (post-acute ischemic injury)	Spatial transcriptomics reveals microglial subclusters stratified across stroke core-penumbra, defining dichotomous neuroimmunity (ICAM1-pro-inflammatory vs. IPAM-preservation) and enabling lesion-targeted immunomodulation therapy.	Li H et al., 2023 [189]

Cerebral organoids, derived from human pluripotent stem cells (hPSCs), have emerged as powerful tools for simulating the complex biology of neurodegenerative diseases. These 3D cultures closely mimic the architecture and cellular diversity of the human brain, enabling long-term intercellular interactions and the retention of pathological protein aggregates, hallmarks of NDDs. For instance, organoid models of Alzheimer’s disease derived from patient iPSCs or gene-edited cells recapitulate key pathological features, including amyloid-β accumulation and tau phosphorylation [139, 140]. Similarly, phosphorylated α-synuclein aggregates have been observed in organoid models of Parkinson’s disease carrying mutations in the LRRK2 gene [141]. These models offer a controlled environment to study early events in neurodegeneration, free from age-related secondary complications, providing a unique opportunity to unravel disease mechanisms at their inception.

The integration of spatial omics technologies, particularly spatial transcriptomics, with cerebral organoids represents a groundbreaking advance in NDD research. Spatial transcriptomics preserves the spatial context of gene expression within tissue-like structures, enabling the identification of region-specific transcriptional changes and cell-cell interactions that drive neurodegenerative pathology. For example, Strooper et al. demonstrated in human and mouse brains that amyloid plaques induce a coordinated multicellular response within the plaque microenvironment, revealing gene co-expression networks associated with neurodegeneration [142]. In addition to AD, other neurodegenerative diseases like multiple sclerosis (MS) and Parkinson’s disease also exhibit abnormal spatial organization of cells and molecules. By integrating spatial transcriptomics with organoid models of these diseases, researchers can uncover the spatial dynamics of cellular dysfunction, identify disease-specific transcriptional signatures, and explore how pathological protein aggregates disrupt neural networks. Such approaches offer critical insights into the interactions among different cell types, such as neurons, glial cells, and immune cells, highlighting pathways that may serve as therapeutic targets.

## Translational applications of spatial omics and cerebral organoids

The combination of spatial omics technologies and cerebral organoids is revolutionizing translational neuroscience by addressing key limitations in preclinical research and advancing drug discovery, personalized medicine, biomarker development, and neurotoxicology. Traditional animal models and 2D cultures, while long relied upon in drug development, often fail to replicate the genetic, molecular, and environmental complexities of human neurological diseases. This has contributed to high failure rates in clinical trials, with only 6–7% of preclinical drugs successfully transitioning to approval due to issues of inefficacy and toxicity [143]. The limitations of these conventional models underscore the urgent need for innovative, human-relevant systems to bridge the translational gap.

Cerebral organoids, derived from human-induced pluripotent stem cells (iPSCs), offer a powerful solution by mimicking the three-dimensional architecture, cellular heterogeneity, and genetic complexity of the human brain. They enable accurate modeling of disease pathology, therapeutic responses, and neurodevelopmental processes, providing a physiologically relevant platform for preclinical testing. Recent regulatory advancements, such as the FDA Modernization Act 2.0, have accelerated the adoption of organoids in preclinical research by recognizing them as alternatives to animal models [144].

The integration of spatial omics technologies further elevates the utility of cerebral organoids by preserving spatial context while profiling molecular changes at cellular resolution. Unlike bulk analysis, which averages signals across tissues, spatial omics—such as spatial transcriptomics, proteomics, and metabolomics—maps gene expression, protein localization, and metabolic activity within organoid structures. This is particularly valuable for neurological diseases, where region-specific variations in molecular activity can drive pathology. For example, spatial transcriptomics in Alzheimer’s disease models has identified dysregulated gene networks associated with amyloid-β and tau deposition, while spatial proteomics has revealed protein-protein interactions linked to synaptic dysfunction [145]. These insights allow researchers to design targeted therapeutics that address disease mechanisms with greater precision, improving efficacy while minimizing off-target effects [146].

In the realm of personalized medicine, patient-specific cerebral organoids, generated from iPSCs, offer the ability to model individual variability in disease presentation and treatment response. Integrating spatial omics technologies enables researchers to pinpoint region-specific disruptions in gene expression, epigenetic regulation, or metabolic pathways unique to each patient. For instance, spatial transcriptomics applied to epilepsy models can identify molecular drivers of neuronal hyperexcitability, informing the development of patient-tailored interventions. These platforms also allow testing of therapies—such as small molecules or gene-editing tools—directly on patient-derived organoids, optimizing treatment strategies and minimizing adverse effects.

The combination of spatial omics and cerebral organoids also holds promise for biomarker discovery. Spatial metabolomics has uncovered early metabolic shifts in Huntington’s disease models, offering potential indicators of disease progression [147], while spatial epigenomics has identified region-specific chromatin modifications in Alzheimer’s disease, providing molecular signatures for early diagnosis and patient stratification. Technologies like spatial-CUT&Tag enable high-resolution mapping of histone modifications and regulatory elements, offering insights into the epigenetic control of brain development and disease progression [73]. These biomarkers improve the precision of clinical trials and facilitate the monitoring of treatment efficacy, ensuring that therapeutic interventions effectively target key molecular pathways.

In neurotoxicology and safety assessments, the combined use of cerebral organoids and spatial omics addresses the shortcomings of traditional toxicity models, which often fail to predict the effects of environmental toxins or drugs on human brain tissues. Cerebral organoids provide a 3D, human-specific platform that models complex neural architectures and cellular interactions. Spatial omics adds a layer of precision by mapping how toxins or drugs affect specific brain regions, disrupt neural circuits, and alter cellular metabolism. For instance, spatial transcriptomics can reveal gene expression changes associated with cellular stress, while spatial metabolomics can identify localized metabolic disruptions indicative of toxicity. This approach enhances the accuracy of toxicity assessments, helping to identify vulnerable brain regions, assess dose-dependent responses, and evaluate long-term therapeutic impacts.

In summary, the integration of spatial omics and cerebral organoids transforms the study of neurological diseases by offering a high-resolution, human-relevant platform for preclinical research. This combination enables precise modeling of disease mechanisms, accelerates biomarker discovery, enhances personalized medicine, and improves neurotoxicity testing. By bridging the gap between preclinical findings and clinical success, this synergistic approach holds immense potential to advance the development of safer and more effective therapies for neurological disorders.

## Conclusions

Spatial omics techniques have revolutionized neuroscience by offering a groundbreaking approach to understanding the nervous development in its spatially resolved molecular and cellular context. Human induced pluripotent stem cell (iPSC)-derived cerebral organoids present a promising alternative, providing a human-specific, patient-relevant platform to study neural development and disease. When combined with spatial omics technologies—such as spatial transcriptomics, proteomics, metabolomics, and epigenomics—cerebral organoids enable a deeper exploration of cell types, neuronal circuitry, and molecular pathways in both healthy and diseased states of the brain. This powerful synergy is poised to advance our understanding of neurodevelopmental disorders and catalyze the development of effective therapeutic strategies.

The integration of spatial omics and cerebral organoids, while transformative, is not without challenges [148]. Spatial omics, for instance, faces difficulties in associating signals with individual cells, particularly in complex neural tissues where glial cells, neurons, and other cell types are closely interwoven. High-resolution spatial mapping requires precise separation of cellular signals, a feat that remains technically demanding [149]. Additionally, scalability presents another hurdle. Generating spatially resolved molecular data across extensive cerebral organoid models at cellular and subcellular resolutions demands high-throughput techniques capable of capturing both depth and breadth. Despite its promise, spatial transcriptomics often struggles with insufficient coverage for low-abundance transcripts and technical noise that can obscure spatial resolution [150]. These limitations can result in an incomplete or skewed understanding of the spatial gene expression landscape, particularly in tissues as intricate as the brain.

Cerebral organoids, while offering unprecedented insights, also face inherent limitations. The reproducibility of organoids remains a concern, as variability in cell type composition and spatial organization can hinder the reliability of findings [151]. Additionally, achieving the maturity and cellular complexity required to model late-onset neurological diseases remains a significant. Most organoids currently represent early developmental stages, limiting their application for studying neurodevelopmental diseases that manifest in adulthood challenge [152]. Advances in bioengineering, such as vascularization and co-culture systems, are critical for promoting organoid maturation and enabling long-term studies of disease progression. Moreover, as cerebral organoids become more complex and biologically representative of human brain tissues, ethical considerations surrounding their use intensify [153–155]. Questions regarding the moral status of organoids, consent for their creation and use, ownership, and post-research handling must be addressed to ensure responsible and ethical progress in this field.

The integration of spatial omics technologies with cerebral organoids represents a transformative advance in neuroscience, providing a powerful platform to map the spatial and molecular dynamics of the brain development. By precisely identifying gene expression, protein interactions, metabolic disruptions, and epigenetic changes within the 3D structure of organoids, this approach offers critical insights into neurological disorders such as neurodevelopmental disorder, ALS and ASD. However, current applications remain limited, with much of the existing data still derived from traditional animal models. To fully realize its potential, more systematic efforts are needed to integrate spatial omics with cerebral organoids, generating data that can be compared to real-world clinical findings. Such efforts will enhance the accuracy of neurodevelopmental disease modeling, improve reproducibility, and advance clinical applications, ultimately accelerating precision therapies, biomarker discovery, and drug development for pediatric neurological diseases.

## Data Availability

No datasets were generated or analysed during the current study.
